# Reduction of Physical Activity Levels During the COVID-19 Pandemic Might Negatively Disturb Sleep Pattern

**DOI:** 10.3389/fpsyg.2020.586157

**Published:** 2020-12-10

**Authors:** Tiego A. Diniz, Diego G. D. Christofaro, William R. Tebar, Gabriel G. Cucato, João Paulo Botero, Marilia Almeida Correia, Raphael M. Ritti-Dias, Mara C. Lofrano-Prado, Wagner L. Prado

**Affiliations:** ^1^Department of Cell and Developmental Biology, Institute of Biomedical Sciences, University of São Paulo, São Paulo, Brazil; ^2^Department of Physical Education, Universidade Estadual Paulista (UNESP), Presidente Prudente, Brazil; ^3^Department of Sport, Exercise and Rehabilitation, Northumbria University, Newcastle, United Kingdom; ^4^Human Movement Science and Rehabilitation Graduation Program, Federal University of São Paulo, Santos, Brazil; ^5^Graduate Program in Medicine, Universidade Nove de Julho, São Paulo, Brazil; ^6^Graduate Program in Rehabilitation Sciences, Universidade Nove de Julho, São Paulo, Brazil; ^7^University of São Paulo, São Paulo, Brazil; ^8^California State University, San Bernardino, San Bernardino, CA, United States

**Keywords:** sleep, COVID-19, physical activity, exercise, health

## Abstract

**Background:**

The outbreak of novel coronavirus disease 2019 (COVID-19) has caused a global panic and public concern due to its mortality ratio and lack of treatments/vaccines. Reduced levels of physical activity have been reported during the outbreak, affecting the normal daily pattern.

**Objective:**

To investigate (i) the relationship of physical activity level with sleep quality and (ii) the effects of reduction physical activity levels on sleep quality.

**Methods:**

A Google form was used to address personal information, COVID-19 personal care, physical activity, and mental health of 1,907 adult volunteers. Binary logistic regression was used to verify the association of physical activity parameters and sleep quality.

**Results:**

Insufficient physical activity levels were a risk factor to have disturbed sleep pattern [OR: 1.28, 95% CI (1.01–1.62)]; however, when the BMI was added to the analysis, there was no more statistical difference [OR: 1.23, 95% CI (0.96–1.57)]. On the other hand, we found that the reduction of physical activity levels was associated with negative changes in sleep quality [OR: 1.73, 95% CI (1.37–2.18)], regardless all the confounders [OR: 1.30, 95% CI (1.01–1.68)], unless when feeling of depression was added in Model 6 [OR: 1.28, 95% CI (0.99–1.66)].

**Conclusion:**

Disruption in daily physical activity routine, rather than physical activity level, negatively influences sleep quality during the COVID-19 quarantine.

## Introduction

The outbreak of novel coronavirus disease 2019 (COVID-19) began on December 2019, induced by severe acute respiratory syndrome coronavirus 2 (SARS-CoV-2), causing a global panic and public concern because of its lethality (Rothan and Byrareddy, 2020). This novel virus rapidly spread worldwide and infected more than 37 million people with a ratio of death around 2.8% (Johns Hopkins University and Medicine, 2020), becoming on 11th March a pandemic, according to World Health Organization (WHO).

Since then, most of the countries advocated for a quarantine period ranging from social distancing to self-isolation at home in order to keep the virus from spreading. Due to neither treatment nor vaccine available^1^, this approach is still the best option to avoid further virus spread. On the other hand, quarantine status was found to negatively affect behavior in general, including but not limited to physiological stress, sleep pattern, and physical activity practice (Brooks et al., 2020).

In this regard, FitBit^®^ recently released a report about their 30 million users, in which they found a daily reduction in step counts up to 38% in European countries (e.g., Spain), while South and North American countries accounted for a reduction of 15% on average (Fitbit^®^, 2020). Several studies have shown the positive effects of physical activity on sleep quality, and it is considered one of the non-pharmacological interventions to improve sleep quality (Banno et al., 2018); however, the impact of reduced physical activity levels caused by the COVID-19 pandemic on sleep parameters still needs to be investigated. Furthermore, there is a close relationship between low levels of physical activity and sleep disorders, since the former negatively affects body composition, such as increase of body fat, which in turn is a well-known independent risk factor development of several sleep problems (Palm et al., 2015).

Therefore, the aim of this study is to investigate (i) the relationship of physical activity level with sleep quality and (ii) the effects of reduction physical activity levels on sleep quality. Here we hypothesize that quarantine might negatively affect sleep quality due the reduction of physical activity practice.

## Materials and Methods

### Sample and Ethics

This survey research was conducted in Brazil between May 05th and 17th 2020. Participants were invited through social media to answer an online questionnaire, and the inclusion criteria were age higher than 18 years and to respond all questions of the survey. This study was approved by the Universidade Nove de Julho’ Ethics Committee before data collection (CAAE #30890220.4.0000.5511). Participants did not identify themselves, and their answers were only included in the sample if they authorized it before the protocol started. All procedures follow the national legislation and the Declaration of Helsinki. The research design and characteristics of the sample have been previously described (Lofrano-Prado et al., 2020).

### Procedures

After the ethics approval, a questionnaire (in Portuguese) in Google Forms was presented to participants with 70 questions divided into 7 domains: (a) personal information; (b) COVID-19 personal care; (c) physical activity; (d) eating behavior; (e) health risk habits; (f) mental health; and (g) overall health.

For the purpose of the present study, only some questions of some domains were selected: (a) personal information, (b) COVID-19 personal care, (c) physical activity, and (f) mental health. The instrument was developed for senior researchers with Ph.D. in different areas (Public Health, Science, Nutrition, Physiology, Human Movement Science, Neuroscience, and Behavior). In the following, we present the questions used in the present analysis.

Sleep quality: from this domain, changes in sleep quality were identified by the following: “Due to the COVID-19, are you feeling lower sleep quality?” (possible answers: “no,” “a little,” “sometimes,” “very often,” and “always”). Participants were classified as “decreased sleep quality” group if they answered “a little,” “sometimes,” “very often,” and “always.”

Personal information: from this domain, three self-reported information were collected: 1—sex (possible answers: “Woman or Man”); 2—date of birthday (DD/MM/YYYY); and 3—educational level (“elementary,” “high school,” “undergraduate,” and “graduate”). In this domain, it was also questioned “What is your weight (in kilogram)?” and “What is your height (in centimeters)?” as open questions.

Social isolation: “How long have you experienced social isolation?” Participants who responded 15 or more days were classified as socially isolated.

Physical activity: participants were asked about the following: (a) How many times are you exercising a week? (possible answers: none to 7 days a week); (b) For how long are you exercising? (possible answers: “none”; “less than 30 min”; “between 30 and 60 min”; and “more than 60 min”); (c) For how long have you engaged in this physical activity? (possible answer: “less than 1 month”; “between 1 and 3 months”; “between 3 and 6 months”; “more than 6 months,” and; “I am not exercising”); (d) What is the intensity of the physical activity? (possible answers: “low—i.e., to bathe, to shave, to drive, wash the dishes, and make the bed”; “medium/moderate—i.e., gardening, play volleyball, water aerobics, pedal, and brisk walking”; “high—i.e., climb stairs, swimming, jump rope, play soccer, running”; and “I am not exercising”).

Based on these responses, time spent during each exercise session during the week was multiplied by the number of days spent exercising each week. Those that reached 150 min or more of moderate-vigorous physical activity (MVPA) were considered “physically active” whereas those that fell below this threshold were classified as “inactive.” In this domain, it was also questioned “How much has the COVID-19 pandemic interfered with your daily physical activity habits?” (possible answers: “none,” “a little,” “a lot,” and “I do not exercise”). Participants who answered “a little” was classified as not having impact on physical activity due to COVID-19 and those who answered a “lot” were classified as having an impact on physical activity due to COVID-19. These participants who answered “I do not exercise” were not considered in the analysis.

Mental health: Feeling of anxiety and depression were assessed by the questions “Due to the COVID-19, are you feeling anxious?” and “Due to the COVID-19, are you feeling depressed?” (possible answers for each question were “no,” “a little,” “sometimes,” “very often,” and “always”). The participants’ responses were dichotomized into i) not having anxiety and/or depression (“no,” “a little,” and “sometimes”) and having anxiety and/or depression (“very often” and “always”).

### Statistical Analysis

The sample characterization variables were presented as mean and standard deviation comparing between participants who did not feel any change in sleep quality with those who felt the impact of sleep quality in the quarantine by means of the Student’s *t* test for independent samples. The association of sleep quality with the practice of physical activity during the pandemic and the impact of the pandemic on physical activity were achieved by binary logistic regression (Model 1: crude model; Model 2: Model 1 + adjusted by sex, age, and education level; Model 3: Model 2 + adjusted by BMI and Model 4: Model 3 adjusted by social isolation). Analyses were performed using the statistical software SPSS (version 13.0), and *p* < 0.05 was used as a statistically significant reference value. The polychoric correlation matrix was performed in the STATA statistical package.

## Results

The sample of the present study was composed of 1,874 adults with an average age of 38.30 (±13.09) years. The prevalence of decreased sleep quality during quarantine was 26.1%. In all, 514 participants (27.4%) were classified as physically active during quarantine (MVPA ≥ 150 min per week) and approximately 56.2% of participants reported that COVID-19 had an impact on physical activity. Table 1 shows the comparison between normal and disturbed sleep quality groups. Disturbed sleep group was younger and less physically active during the week (*p* < 0.05).

**TABLE 1:** Characteristics of the sample dichotomized by sleep changes.

	Normal sleep quality	Disturbed sleep quality	*p*-value
Age (years)	39.72 (13.50)	34.29 (10.90)	**<0.001**
Weight (kg)	73.63 (16.45)	73.36 (16.77)	0.755
Height (cm)	168.78 (9.28)	168.01 (8.91)	0.114
BMI (kg)	25.65 (4.38)	25.83 (4.74)	0.463
MVPA (min/week)	90.77 (93.32)	76.25 (91.89)	**0.004**

*BMI, body mass index; MVPA, moderate to vigorous physical activity. Bold values denote statistical significance at the p < 0.05 level.*

In Table 2 is shown the association of insufficient MVPA levels according to ACMS with sleep quality. We found that in the crude analysis (model 1) and adjusted by confounders (model 2), such as sex, age, and education practice, less than 150 min of MVPA has a detrimental effect upon sleep quality. Nonetheless, when the analyses were adjusted for BMI, social isolation, feeling of anxiety, and feeling of depression, the models did not reach statistical significance (model 3 to 6).

**TABLE 2:** Association of insufficient MVPA and changes in quality of sleep in adults during COVID-19.

	Impaired sleep quality during COVID-19		
	OR	95% CI	*p*-value
**Insufficient MVPA**			
Model 1	1.28	1.01–1.62	**0.044**
Model 2	1.29	1.01–1.65	**0.040**
Model 3	1.23	0.96–1.57	0.108
Model 4	1.21	0.94–1.56	0.133
Model 5	1.17	0.90–1.52	0.247
Model 6	1.16	0.88–1.51	0.289

*MVPA, Moderate/Vigorous physical activity; Model 1, crude model; Model 2, model 1 + adjusted by sex, age, and education level; Model 3, model 2 + adjusted by BMI; Model 4, model 3 adjusted by social isolation; Model 5, model 4 adjusted by feeling of anxiety; Model 6, model 5 adjusted by feeling of depression. Bold values denote statistical significance at the p < 0.05 level.*

Table 3 shows the effects of reduction of physical activity levels upon sleep quality during the quarantine period. Without addition of confounders, it was found that decrease in physical activity practice increased up to 1.72 times the risk of sleep disturbances. This relationship dropped to 1.53 times when the confounders of sociodemographic variables, BMI, social isolation, and feeling of anxiety were added to the analysis. After the adjustment for feeling of depression, the association between reduction in physical activity level and impaired sleep quality lost statistical significance.

**TABLE 3:** Association of the impact of COVID-19 in the physical activity practice and changes in quality of sleep in adults.

	Impaired sleep quality during COVID-19		
	OR	95% CI	*p*-value
**Reduction of PA by COVID-19**			
Model 1	1.73	1.37–2.18	**<0.001**
Model 2	1.69	1.33–2.14	**<0.001**
Model 3	1.58	1.25–2.01	**<0.001**
Model 4	1.54	1.21–1.96	**<0.001**
Model 5	1.30	1.01–1.68	**0.043**
Model 6	1.28	0.99–1.66	**0.062**

*PA, physical activity; Model 1, crude model; Model 2, model 1 + adjusted by sex, age, and education level; Model 3, model 2 + adjusted by BMI; Model 4, model 3 adjusted by social isolation; Model 5, model 4 adjusted by feeling of anxiety; Model 6, model 5 adjusted by feeling of depression. Bold values denote statistical significance at the p < 0.05 level.*

## Discussion

In this study, we found that rather than physical activity level, the reduction of physical exercise practice during the COVID-19-induced quarantine increased the risk of sleep disturbance up to 1.5 times regardless of potential confounders.

The implications of COVID-19-induced confinement can be severe upon neuromuscular, cardiovascular, and metabolic health and sleep quality if physical activity levels decrease abruptly. For example, acute reduction in the physical activity levels lead to impaired insulin sensitivity and glucose handling, decreases in lean body mass and strength, and increases in visceral adiposity (Narici et al., 2020). Furthermore, adults who removed structured physical exercise for one week and, therefore, decreased their physical activity levels experienced an impairment in sleep quality (Edwards and Loprinzi, 2017). Long-term sleep disturbances have also been associated with impaired metabolic health (Gabriel and Zierath, 2019).

Here we found that the relationship of insufficient physical activity levels and sleep quality is affected by BMI. Obesity *per se* is a risk factor for sleep disorders. Weight gain across time is an independent risk factor for developing a range of sleep problems and daytime sleepiness (Palm et al., 2015). Otherwise, the reduction of physical activity levels during the COVID-19 outbreak increased 1.5 times the odds to decrease sleep quality regardless confounders, such as BMI, showing the importance to keep physical activity practice during this outbreak.

On the other hand, physical activity practice may improve metabolic health and sleep quality (Narici et al., 2020). In a recent meta-analysis, Banno et al. (2018) showed that the exercised group decreased up to 3 points in the Pittsburgh Sleep Quality Index, a questionnaire to address sleep quality, and 3.22 points in the Insomnia Severity Index when compared with the control group. In fact, a slight increase in daily physical levels is an effective strategy to improve sleep duration and decrease sleep latency (Hori et al., 2016).

The association between physical activity and impaired sleep quality due to COVID-19 is lacking when adjusted for depression feelings. The bidirectional association between sleep disorders and depression has been previously reported (Fang et al., 2019). Prevalence of depression is supposed to be 7 times higher during the COVID-19 outbreak than the previous global estimate (Bueno-Notivol et al., 2020). In this sense, it is possible that reduction in physical activity levels due to the COVID-19 outbreak has not been so strong as to override the association between depression and sleep quality in the subjects.

Sleep is a complex behavior that can be affected by a diversity of variables. Physical exercise has been described as a zeitgeber, a rhythmically external or environmental cue that acts in the regulation of the body’s circadian rhythms (Gabriel and Zierath, 2019); therefore, we hypothesize that reduction of physical activity levels during the quarantine period might be affect the internal body clock and, therefore, impair sleep quality.

In order to maintain physical activity levels during the COVID-19-induced quarantine, home-based interventions might be a strategy. Several studies have shown that this kind of intervention is as effective as supervised ones to improve the body composition, metabolic profile, and physical fitness in a broad population (Emerenziani et al., 2018). Recently, the European CBT-I Academy published practical recommendations for improving sleep problems during the COVID-19 outbreak, in which they recommend physical exercise, especially in the daylight, as a tool to sleep better (Altena et al., 2020).

The effects of home-based exercise programs have been reported on sleep quality parameters in the literature. Cheville et al. (2013) conducted a randomized controlled trial to assess sleep quality in cancer patients who underwent a home-based exercise routine (strength and walking exercises) and found an improvement in sleep quality in the exercised group. Similarly, Tang et al. (2010) found that in cancer patients who performed home-based exercises the quality of sleep improved when compared to usual-care patients. Moreover, in patients with chronic kidney disease, Aoike et al. (2015) found that a home-based exercise program was able to decrease up to 3.3 points in the Pittsburgh Sleep Quality Index Questionnaire.

The limitations of this article should be mentioned: the use of an electronic-based questionnaire, which could represent a selection bias, and the use of a non-validated questionnaire, which could represent a non-exact method to estimate the outcomes. Some strengths should also be highlighted, such as the inclusion of other domains in the same questionnaire, the use of Likert scales, and the sample size.

In practical terms, since quarantine has disrupted normal daily physical activity events and impaired some people to keep doing gym and outside workout, home-based physical exercise is indeed recommended, preferably guided by a coach, in order to improve sleep quality.

Therefore, we showed that the disruption in daily physical activity routine rather than physical activity level negatively influences sleep quality. Therefore, public policies should be created in order to stimulate the practicing of physical exercise in a home-based approach.

## Data Availability Statement

The datasets presented in this study can be found in online repositories. The names of the repository/repositories and accession number(s) can be found in the article/Supplementary Material.

## Ethics Statement

The studies involving human participants were reviewed and approved by Universidade Nove de Julho CAAE #30890220.4.0000.5511. Before the beginning of the protocol, all participants consented to their participation.

## Author Contributions

MC, RR-D, and GC: data collection. TD, DC, GC, WT, ML-P, and JB: study design. TD, DC, WT, GC, ML-P, and JB: data analysis. TD, DC, GC, JB, ML-P, and WT: manuscript draft. All authors contributed to the revision.

## Conflict of Interest

The authors declare that the research was conducted in the absence of any commercial or financial relationships that could be construed as a potential conflict of interest.

## Footnotes

1 clinicaltrials.gov
