# Supplementary material for: Reduction of Physical Activity Levels During the COVID-19 Pandemic Might Negatively Disturb Sleep Pattern
Source: Front Psychol. 2020 Dec 10;11:586157. doi: 10.3389/fpsyg.2020.586157 (PMC7793775; doi:10.3389/fpsyg.2020.586157)
Supplement: Supplementary file 1 [file Data_Sheet_1.PDF]

```
. polychoricpca  Sexo_DIC Intrucao_DIC Isolamento_social_DIC
Impacto_pandemia_AF IMC_DIC Idade Frequencia_semanal_pratica_AF_co Du
> ração_cada_sessão_em_minuto Tempo_de_Pratica_DIC Intensidade_AF_DIC
qualidade_sono_piorou Sentir_Ansioso Sentiu_deprimido, pw

Polychoric correlation matrix

                                                                 Sexo_DIC
Intrucao_DIC
                           Sexo_DIC                                     1
                       Intrucao_DIC                              .0726245
1
              Isolamento_social_DIC                             .00875398
.09872475
                Impacto_pandemia_AF                            -.03438635
.0285771
                            IMC_DIC                            -.33091058
.03594243
                              Idade                            -.09140694
.19946191
   Frequencia_semanal_pratica_AF_co                            -.09516527
.02618624
Duração_cada_sessão_em_minutos_c                            -.13538627
.05175555
               Tempo_de_Pratica_DIC                             -.0876319
.05825524
                 Intensidade_AF_DIC                            -.10347748
.05213433
              qualidade_sono_piorou                             .10421147
-.02848711
                     Sentir_Ansioso                             .29072465
.02951049
                   Sentiu_deprimido                             .19434627
-.06247121

                                                    Isolamento_social_DIC
Impacto_pandemia_AF
              Isolamento_social_DIC                                     1
                Impacto_pandemia_AF                             .03138995
1
                            IMC_DIC                             -.0076711
.06487175
                              Idade                             .03340665
-.06130818
   Frequencia_semanal_pratica_AF_co                             .05412578
-.17595814
Duração_cada_sessão_em_minutos_c                            -.05568224
-.08204852
               Tempo_de_Pratica_DIC                             -.0083636
-.08873127
                 Intensidade_AF_DIC                             .03862924
-.04308925
              qualidade_sono_piorou                            -.00627765
.18829416
                     Sentir_Ansioso                             .10274607
.19811321
```

```
                Sentiu_deprimido                     .05300667
.14144191

                                                       IMC_DIC
Idade
                         IMC_DIC                             1
                           Idade                     .27562184
1
   Frequencia_semanal_pratica_AF_co                 -.16203853
.02030118
Duração_cada_sessão_em_minutos_c                 -.11352915
.04729075
             Tempo_de_Pratica_DIC                   -.16119124
.04893087
               Intensidade_AF_DIC                   -.24392695
-.15777874
           qualidade_sono_piorou                     .04295564
-.18568609
                  Sentir_Ansioso                    -.00531667
-.23850531
                Sentiu_deprimido                    -.00014079
-.19222414

                                  Frequencia_semanal_pratica_AF_co
Duração_cada_sessão_em_minutos_c
   Frequencia_semanal_pratica_AF_co                              1
Duração_cada_sessão_em_minutos_c                  .64048415
1
             Tempo_de_Pratica_DIC                    .62585592
.65888616
               Intensidade_AF_DIC                    .68015395
.66591567
           qualidade_sono_piorou                    -.13876613
-.12377933
                  Sentir_Ansioso                     -.1071127
-.10119613
                Sentiu_deprimido                    -.14253991
-.12727902

                                              Tempo_de_Pratica_DIC
Intensidade_AF_DIC
             Tempo_de_Pratica_DIC                                1
               Intensidade_AF_DIC                    .74719338
1
           qualidade_sono_piorou                    -.09684826
-.05718776
                  Sentir_Ansioso                    -.10081815
-.02707789
                Sentiu_deprimido                    -.11499319
-.10148461

                                             qualidade_sono_piorou
Sentir_Ansioso
           qualidade_sono_piorou                                 1
                  Sentir_Ansioso                     .48934359
1
                Sentiu_deprimido                     .49111111
.68661667
```

Principal component analysis

k	Eigenvalues	Proportion explained	Cum. explained
1	3.261366	0.250874	0.250874
2	2.262144	0.174011	0.424885
3	1.373689	0.105668	0.530554
4	1.212174	0.093244	0.623798
5	0.973813	0.074909	0.698707
6	0.930988	0.071614	0.770321
7	0.724481	0.055729	0.826050
8	0.564534	0.043426	0.869476
9	0.498612	0.038355	0.907831
10	0.372003	0.028616	0.936447
11	0.337362	0.025951	0.962397
12	0.286927	0.022071	0.984469
13	0.201906	0.015531	1.000000

.
