# Supplementary material for: Reduction of Physical Activity Levels During the COVID-19 Pandemic Might Negatively Disturb Sleep Pattern
Source: Front Psychol. 2020 Dec 10;11:586157. doi: 10.3389/fpsyg.2020.586157 (PMC7793775; doi:10.3389/fpsyg.2020.586157)
Supplement: Supplementary file 2 [file Data_Sheet_2.PDF]

```
. polychoric  Sexo_DIC Intrucao_DIC Isolamento_social_DIC
Impacto_pandemia_AF IMC_DIC Idade Frequencia_semanal_pratica_AF_co Duraç
> ão_cada_sessão_em_minuto Tempo_de_Pratica_DIC Intensidade_AF_DIC
qualidade_sono_piorou Sentir_Ansioso Sentiu_deprimido, pw  verb

Variables :  Sexo_DIC Intrucao_DIC
Type :       polychoric
Rho         = .0726245
S.e.        = .0330582
Goodness of fit tests:
Pearson G2 = 10.326479, Prob( >chi2(2)) = .00572313
LR X2      = 10.338128, Prob( >chi2(2)) = .00568989

Variables :  Sexo_DIC Isolamento_social_DIC
Type :       polychoric
Rho         = .00875398
S.e.        = .05285231
Goodness of fit tests:
Pearson G2 = ., Prob( >chi2(.)) = .
LR X2      = ., Prob( >chi2(.)) = .

Variables :  Sexo_DIC Impacto_pandemia_AF
Type :       polychoric
Rho         = -.03438635
S.e.        = .03542563
Goodness of fit tests:
Pearson G2 = 7.722282, Prob( >chi2(1)) = .00545434
LR X2      = 7.498772, Prob( >chi2(1)) = .00617411

Variables :  Sexo_DIC IMC_DIC
Type :       polychoric
Rho         = -.33091058
S.e.        = .03063339
Goodness of fit tests:
Pearson G2 = 14.003874, Prob( >chi2(1)) = .00018243
LR X2      = 14.087557, Prob( >chi2(1)) = .00017449

Variables :  Sexo_DIC Idade
Type :       polyserial
Rho         = -.09140694
S.e.        = .02868883

Variables :  Sexo_DIC Frequencia_semanal_pratica_AF_co
Type :       polychoric
Rho         = -.09516527
S.e.        = .02963276
Goodness of fit tests:
Pearson G2 = 4.0730763, Prob( >chi2(6)) = .66678771
LR X2      = 4.0928899, Prob( >chi2(6)) = .66410737

Variables :  Sexo_DIC Duração_cada_sessão_em_minutos_c
Type :       polychoric
Rho         = -.13538627
S.e.        = .03243836
Goodness of fit tests:
Pearson G2 = 2.1189143, Prob( >chi2(1)) = .14548965
LR X2      = 2.1143987, Prob( >chi2(1)) = .14591936
```

```
Variables :  Sexo_DIC Tempo_de_Pratica_DIC
Type :       polychoric
Rho        = -.0876319
S.e.       = .03160269
Goodness of fit tests:
Pearson G2 = 3.2719084, Prob( >chi2(3)) = .35157165
LR X2      = 3.2854735, Prob( >chi2(3)) = .34966958

Variables :  Sexo_DIC Intensidade_AF_DIC
Type :       polychoric
Rho        = -.10347748
S.e.       = .03144992
Goodness of fit tests:
Pearson G2 = 2.160123, Prob( >chi2(2)) = .33957465
LR X2      = 2.1637195, Prob( >chi2(2)) = .33896454

Variables :  Sexo_DIC qualidade_sono_piorou
Type :       polychoric
Rho        = .10421147
S.e.       = .03085112
Goodness of fit tests:
Pearson G2 = 1.2478507, Prob( >chi2(3)) = .74155207
LR X2      = 1.2482434, Prob( >chi2(3)) = .7414583

Variables :  Sexo_DIC Sentir_Ansioso
Type :       polychoric
Rho        = .29072465
S.e.       = .02797136
Goodness of fit tests:
Pearson G2 = 2.6783189, Prob( >chi2(3)) = .44392435
LR X2      = 2.6833102, Prob( >chi2(3)) = .44307101

Variables :  Sexo_DIC Sentiu_deprimido
Type :       polychoric
Rho        = .19434627
S.e.       = .03080312
Goodness of fit tests:
Pearson G2 = 1.4300174, Prob( >chi2(2)) = .48918785
LR X2      = 1.4340799, Prob( >chi2(2)) = .4881952

Variables :  Intrucao_DIC Isolamento_social_DIC
Type :       polychoric
Rho        = .09872475
S.e.       = .04737611
Goodness of fit tests:
Pearson G2 = .25364399, Prob( >chi2(2)) = .88089046
LR X2      = .25631718, Prob( >chi2(2)) = .87971385

Variables :  Intrucao_DIC Impacto_pandemia_AF
Type :       polychoric
Rho        = .0285771
S.e.       = .03116234
Goodness of fit tests:
Pearson G2 = 16.80412, Prob( >chi2(5)) = .00488667
LR X2      = 20.060016, Prob( >chi2(5)) = .00121773

Variables :  Intrucao_DIC IMC_DIC
```

```
Type :       polychoric
Rho         = .03594243
S.e.        = .03055959
Goodness of fit tests:
Pearson G2 = 18.012395, Prob( >chi2(5)) = .00293091
LR X2      = 17.782373, Prob( >chi2(5)) = .00323189

Variables :  Intrucao_DIC Idade
Type :       polyserial
Rho         = .19946191
S.e.        = .0292336

Variables :  Intrucao_DIC Frequencia_semanal_pratica_AF_co
Type :       polychoric
Rho         = .02618624
S.e.        = .02825073
Goodness of fit tests:
Pearson G2 = 38.751479, Prob( >chi2(20)) = .00715798
LR X2      = 33.127309, Prob( >chi2(20)) = .03267099

Variables :  Intrucao_DIC Duração_cada_sessão_em_minutos_c
Type :       polychoric
Rho         = .05175555
S.e.        = .03057842
Goodness of fit tests:
Pearson G2 = 22.314113, Prob( >chi2(5)) = .00045628
LR X2      = 22.715262, Prob( >chi2(5)) = .0003826

Variables :  Intrucao_DIC Tempo_de_Pratica_DIC
Type :       polychoric
Rho         = .05825524
S.e.        = .03010225
Goodness of fit tests:
Pearson G2 = 19.844245, Prob( >chi2(11)) = .04752303
LR X2      = 12.935279, Prob( >chi2(11)) = .29757662

Variables :  Intrucao_DIC Intensidade_AF_DIC
Type :       polychoric
Rho         = .05213433
S.e.        = .02956865
Goodness of fit tests:
Pearson G2 = 24.478659, Prob( >chi2(8)) = .0019042
LR X2      = 21.618036, Prob( >chi2(8)) = .00567483

Variables :  Intrucao_DIC qualidade_sono_piorou
Type :       polychoric
Rho         = -.02848711
S.e.        = .0281235
Goodness of fit tests:
Pearson G2 = 10.546556, Prob( >chi2(11)) = .48199445
LR X2      = 8.482472, Prob( >chi2(11)) = .66954314

Variables :  Intrucao_DIC Sentir_Ansioso
Type :       polychoric
Rho         = .02951049
S.e.        = .0276296
Goodness of fit tests:
Pearson G2 = 20.962074, Prob( >chi2(11)) = .03376612
```

```
LR X2      = 16.803871, Prob( >chi2(11)) = .1138124

Variables :  Intrucao_DIC Sentiu_deprimido
Type :       polychoric
Rho        = -.06247121
S.e.       = .02800839
Goodness of fit tests:
Pearson G2 = 10.371062, Prob( >chi2(8)) = .23994175
LR X2      = 7.5071349, Prob( >chi2(8)) = .48303026

Variables :  Isolamento_social_DIC Impacto_pandemia_AF
Type :       polychoric
Rho        = .03138995
S.e.       = .04990728
Goodness of fit tests:
Pearson G2 = 1.6497406, Prob( >chi2(1)) = .19899425
LR X2      = 1.7986948, Prob( >chi2(1)) = .17987037

Variables :  Isolamento_social_DIC IMC_DIC
Type :       polychoric
Rho        = -.0076711
S.e.       = .04790273
Goodness of fit tests:
Pearson G2 = .23489072, Prob( >chi2(1)) = .62792091
LR X2      = .23611179, Prob( >chi2(1)) = .6270286

Variables :  Isolamento_social_DIC Idade
Type :       polyserial
Rho        = .03340665
S.e.       = .04055114

Variables :  Isolamento_social_DIC Frequencia_semanal_pratica_AF_co
Type :       polychoric
Rho        = .05412578
S.e.       = .0444961
Goodness of fit tests:
Pearson G2 = 6.4375953, Prob( >chi2(6)) = .37599446
LR X2      = 6.2118177, Prob( >chi2(6)) = .39988545

Variables :  Isolamento_social_DIC Duração_cada_sessão_em_minutos_c
Type :       polychoric
Rho        = -.05568224
S.e.       = .05127481
Goodness of fit tests:
Pearson G2 = 12.281738, Prob( >chi2(1)) = .00045741
LR X2      = 12.732049, Prob( >chi2(1)) = .00035944

Variables :  Isolamento_social_DIC Tempo_de_Pratica_DIC
Type :       polychoric
Rho        = -.0083636
S.e.       = .04852403
Goodness of fit tests:
Pearson G2 = 7.7537034, Prob( >chi2(3)) = .05138584
LR X2      = 7.6460416, Prob( >chi2(3)) = .0539224

Variables :  Isolamento_social_DIC Intensidade_AF_DIC
Type :       polychoric
Rho        = .03862924
```

```
S.e.       = .04543182
Goodness of fit tests:
Pearson G2 = 1.825683, Prob( >chi2(2)) = .40138208
LR X2      = 1.909737, Prob( >chi2(2)) = .38486274

Variables :  Isolamento_social_DIC qualidade_sono_piorou
Type :       polychoric
Rho        = -.00627765
S.e.       = .04261463
Goodness of fit tests:
Pearson G2 = 4.449931, Prob( >chi2(3)) = .21680004
LR X2      = 4.6392762, Prob( >chi2(3)) = .20019855

Variables :  Isolamento_social_DIC Sentir_Ansioso
Type :       polychoric
Rho        = .10274607
S.e.       = .04250844
Goodness of fit tests:
Pearson G2 = 1.7561291, Prob( >chi2(3)) = .62452794
LR X2      = 1.7891593, Prob( >chi2(3)) = .61729682

Variables :  Isolamento_social_DIC Sentiu_deprimido
Type :       polychoric
Rho        = .05300667
S.e.       = .04599645
Goodness of fit tests:
Pearson G2 = 4.558136, Prob( >chi2(2)) = .10237958
LR X2      = 4.5712255, Prob( >chi2(2)) = .10171172

Variables :  Impacto_pandemia_AF IMC_DIC
Type :       polychoric
Rho        = .06487175
S.e.       = .0318736
Goodness of fit tests:
Pearson G2 = 26.603559, Prob( >chi2(3)) = 7.129e-06
LR X2      = 29.668162, Prob( >chi2(3)) = 1.621e-06

Variables :  Impacto_pandemia_AF Idade
Type :       polyserial
Rho        = -.06130818
S.e.       = .02757223

Variables :  Impacto_pandemia_AF Frequencia_semanal_pratica_AF_co
Type :       polychoric
Rho        = -.17595814
S.e.       = .02575015
Goodness of fit tests:
Pearson G2 = 468.60832, Prob( >chi2(13)) = 6.727e-92
LR X2      = 551.78273, Prob( >chi2(13)) = 1.43e-109

Variables :  Impacto_pandemia_AF Duração_cada_sessão_em_minutos_c
Type :       polychoric
Rho        = -.08204852
S.e.       = .03010459
Goodness of fit tests:
Pearson G2 = 289.49005, Prob( >chi2(3)) = 1.872e-62
LR X2      = 260.305, Prob( >chi2(3)) = 3.862e-56
```

```
Variables :  Impacto_pandemia_AF Tempo_de_Pratica_DIC
Type :       polychoric
Rho        = -.08873127
S.e.       = .02761179
Goodness of fit tests:
Pearson G2 = 438.26138, Prob( >chi2(7)) = 1.472e-90
LR X2      = 592.35178, Prob( >chi2(7)) = 1.08e-123

Variables :  Impacto_pandemia_AF Intensidade_AF_DIC
Type :       polychoric
Rho        = -.04308925
S.e.       = .02751737
Goodness of fit tests:
Pearson G2 = 425.91191, Prob( >chi2(5)) = 7.696e-90
LR X2      = 540.26017, Prob( >chi2(5)) = 1.62e-114

Variables :  Impacto_pandemia_AF qualidade_sono_piorou
Type :       polychoric
Rho        = .18829416
S.e.       = .0286452
Goodness of fit tests:
Pearson G2 = 12.789456, Prob( >chi2(7)) = .07740746
LR X2      = 13.22176, Prob( >chi2(7)) = .06688609

Variables :  Impacto_pandemia_AF Sentir_Ansioso
Type :       polychoric
Rho        = .19811321
S.e.       = .02779701
Goodness of fit tests:
Pearson G2 = 12.509325, Prob( >chi2(7)) = .08500517
LR X2      = 12.370137, Prob( >chi2(7)) = .0890251

Variables :  Impacto_pandemia_AF Sentiu_deprimido
Type :       polychoric
Rho        = .14144191
S.e.       = .02950737
Goodness of fit tests:
Pearson G2 = 17.529768, Prob( >chi2(5)) = .00359731
LR X2      = 18.034067, Prob( >chi2(5)) = .00290401

Variables :  IMC_DIC Idade
Type :       polyserial
Rho        = .27562184
S.e.       = .02367273

Variables :  IMC_DIC Frequencia_semanal_pratica_AF_co
Type :       polychoric
Rho        = -.16203853
S.e.       = .02795842
Goodness of fit tests:
Pearson G2 = 21.041591, Prob( >chi2(13)) = .07211007
LR X2      = 21.342785, Prob( >chi2(13)) = .0664196

Variables :  IMC_DIC Duração_cada_sessão_em_minutos_c
Type :       polychoric
Rho        = -.11352915
S.e.       = .03011311
Goodness of fit tests:
```

```
Pearson G2 = 6.8148205, Prob( >chi2(3)) = .07804024
LR X2      = 6.8281833, Prob( >chi2(3)) = .07758053

Variables :  IMC_DIC Tempo_de_Pratica_DIC
Type :       polychoric
Rho        = -.16119124
S.e.       = .0288385
Goodness of fit tests:
Pearson G2 = 14.082791, Prob( >chi2(7)) = .04972827
LR X2      = 14.333537, Prob( >chi2(7)) = .04555728

Variables :  IMC_DIC Intensidade_AF_DIC
Type :       polychoric
Rho        = -.24392695
S.e.       = .02747798
Goodness of fit tests:
Pearson G2 = 6.7226753, Prob( >chi2(5)) = .24209567
LR X2      = 6.6420946, Prob( >chi2(5)) = .24864747

Variables :  IMC_DIC qualidade_sono_piorou
Type :       polychoric
Rho        = .04295564
S.e.       = .02830971
Goodness of fit tests:
Pearson G2 = 2.9010892, Prob( >chi2(7)) = .89399899
LR X2      = 2.8862008, Prob( >chi2(7)) = .89532617

Variables :  IMC_DIC Sentir_Ansioso
Type :       polychoric
Rho        = -.00531667
S.e.       = .02730434
Goodness of fit tests:
Pearson G2 = 10.647046, Prob( >chi2(7)) = .15477285
LR X2      = 10.677739, Prob( >chi2(7)) = .15330666

Variables :  IMC_DIC Sentiu_deprimido
Type :       polychoric
Rho        = -.00014079
S.e.       = .02930164
Goodness of fit tests:
Pearson G2 = 1.2305841, Prob( >chi2(5)) = .9419098
LR X2      = 1.2275134, Prob( >chi2(5)) = .94221075

Variables :  Idade Frequencia_semanal_pratica_AF_co
Type :       polyserial
Rho        = .02030118
S.e.       = .02478592

Variables :  Idade Duração_cada_sessão_em_minutos_c
Type :       polyserial
Rho        = .04729075
S.e.       = .02654906

Variables :  Idade Tempo_de_Pratica_DIC
Type :       polyserial
Rho        = .04893087
S.e.       = .02492409
```

```
Variables :  Idade Intensidade_AF_DIC
Type :       polyserial
Rho         = -.15777874
S.e.        = .02243074

Variables :  Idade qualidade_sono_piorou
Type :       polyserial
Rho         = -.18568609
S.e.        = .02268425

Variables :  Idade Sentir_Ansioso
Type :       polyserial
Rho         = -.23850531
S.e.        = .02187103

Variables :  Idade Sentiu_deprimido
Type :       polyserial
Rho         = -.19222414
S.e.        = .02339204

Variables :  Frequencia_semanal_pratica_AF_co
Duração_cada_sessão_em_minutos_c
Type :       polychoric
Rho         = .64048416
S.e.        = .01689612
Goodness of fit tests:
Pearson G2 = 352.66827, Prob( >chi2(13)) = 2.130e-67
LR X2      = 313.09127, Prob( >chi2(13)) = 4.363e-59

Variables :  Frequencia_semanal_pratica_AF_co Tempo_de_Pratica_DIC
Type :       polychoric
Rho         = .62585591
S.e.        = .01682731
Goodness of fit tests:
Pearson G2 = 813.63215, Prob( >chi2(27)) = 5.25e-154
LR X2      = 646.8025, Prob( >chi2(27)) = 5.06e-119

Variables :  Frequencia_semanal_pratica_AF_co Intensidade_AF_DIC
Type :       polychoric
Rho         = .68015428
S.e.        = .01502023
Goodness of fit tests:
Pearson G2 = 499.34134, Prob( >chi2(20)) = 3.998e-93
LR X2      = 456.30993, Prob( >chi2(20)) = 3.938e-84

Variables :  Frequencia_semanal_pratica_AF_co qualidade_sono_piorou
Type :       polychoric
Rho         = -.13876613
S.e.        = .0254105
Goodness of fit tests:
Pearson G2 = 40.834962, Prob( >chi2(27)) = .04271451
LR X2      = 43.323727, Prob( >chi2(27)) = .02425585

Variables :  Frequencia_semanal_pratica_AF_co Sentir_Ansioso
Type :       polychoric
Rho         = -.1071127
S.e.        = .02583769
Goodness of fit tests:
```

```
Pearson G2 = 36.508382, Prob( >chi2(27)) = .10461574
LR X2      = 36.06255, Prob( >chi2(27)) = .11393873

Variables :  Frequencia_semanal_pratica_AF_co Sentiu_deprimido
Type :       polychoric
Rho         = -.14253991
S.e.        = .02614426
Goodness of fit tests:
Pearson G2 = 17.540824, Prob( >chi2(20)) = .61762788
LR X2      = 17.802159, Prob( >chi2(20)) = .60043919

Variables :  Duração_cada_sessão_em_minutos_c Tempo_de_Pratica_DIC
Type :       polychoric
Rho         = .65888616
S.e.        = .01726488
Goodness of fit tests:
Pearson G2 = 276.18564, Prob( >chi2(7)) = 7.308e-56
LR X2      = 224.76463, Prob( >chi2(7)) = 6.424e-45

Variables :  Duração_cada_sessão_em_minutos_c Intensidade_AF_DIC
Type :       polychoric
Rho         = .66591567
S.e.        = .01740226
Goodness of fit tests:
Pearson G2 = 234.14696, Prob( >chi2(5)) = 1.381e-48
LR X2      = 216.91558, Prob( >chi2(5)) = 6.802e-45

Variables :  Duração_cada_sessão_em_minutos_c qualidade_sono_piorou
Type :       polychoric
Rho         = -.12377933
S.e.        = .02792901
Goodness of fit tests:
Pearson G2 = 4.4312945, Prob( >chi2(7)) = .728974
LR X2      = 4.3641279, Prob( >chi2(7)) = .73701071

Variables :  Duração_cada_sessão_em_minutos_c Sentir_Ansioso
Type :       polychoric
Rho         = -.10119613
S.e.        = .02845358
Goodness of fit tests:
Pearson G2 = 20.909459, Prob( >chi2(7)) = .00390649
LR X2      = 20.370822, Prob( >chi2(7)) = .00482201

Variables :  Duração_cada_sessão_em_minutos_c Sentiu_deprimido
Type :       polychoric
Rho         = -.12727902
S.e.        = .02895665
Goodness of fit tests:
Pearson G2 = 11.254955, Prob( >chi2(5)) = .04655284
LR X2      = 11.332981, Prob( >chi2(5)) = .04516336

Variables :  Tempo_de_Pratica_DIC Intensidade_AF_DIC
Type :       polychoric
Rho         = .74719338
S.e.        = .01233511
Goodness of fit tests:
Pearson G2 = 462.53552, Prob( >chi2(11)) = 3.089e-92
LR X2      = 370.95782, Prob( >chi2(11)) = 8.843e-73
```

```
Variables :  Tempo_de_Pratica_DIC qualidade_sono_piorou
Type :       polychoric
Rho        = -.09684826
S.e.       = .02667923
Goodness of fit tests:
Pearson G2 = 26.495844, Prob( >chi2(15)) = .03312284
LR X2      = 25.529033, Prob( >chi2(15)) = .04327424

Variables :  Tempo_de_Pratica_DIC Sentir_Ansioso
Type :       polychoric
Rho        = -.10081815
S.e.       = .02684991
Goodness of fit tests:
Pearson G2 = 21.913929, Prob( >chi2(15)) = .11008074
LR X2      = 22.195268, Prob( >chi2(15)) = .10278509

Variables :  Tempo_de_Pratica_DIC Sentiu_deprimido
Type :       polychoric
Rho        = -.11499319
S.e.       = .02734623
Goodness of fit tests:
Pearson G2 = 25.004899, Prob( >chi2(11)) = .00910164
LR X2      = 24.61595, Prob( >chi2(11)) = .01037212

Variables :  Intensidade_AF_DIC qualidade_sono_piorou
Type :       polychoric
Rho        = -.05718776
S.e.       = .02739995
Goodness of fit tests:
Pearson G2 = 12.498839, Prob( >chi2(11)) = .32733754
LR X2      = 12.413663, Prob( >chi2(11)) = .33336585

Variables :  Intensidade_AF_DIC Sentir_Ansioso
Type :       polychoric
Rho        = -.02707789
S.e.       = .02696935
Goodness of fit tests:
Pearson G2 = 13.619205, Prob( >chi2(11)) = .25478491
LR X2      = 13.611192, Prob( >chi2(11)) = .25525898

Variables :  Intensidade_AF_DIC Sentiu_deprimido
Type :       polychoric
Rho        = -.10148461
S.e.       = .02765692
Goodness of fit tests:
Pearson G2 = 10.76706, Prob( >chi2(8)) = .21525043
LR X2      = 10.831445, Prob( >chi2(8)) = .21143391

Variables :  qualidade_sono_piorou Sentir_Ansioso
Type :       polychoric
Rho        = .48934359
S.e.       = .02154428
Goodness of fit tests:
Pearson G2 = 90.603489, Prob( >chi2(15)) = 7.653e-13
LR X2      = 102.91995, Prob( >chi2(15)) = 3.639e-15

Variables :  qualidade_sono_piorou Sentiu_deprimido
```

```
Type :       polychoric
Rho         = .49111111
S.e.        = .02236784
Goodness of fit tests:
Pearson G2 = 43.984624, Prob( >chi2(11)) = 7.312e-06
LR X2      = 44.603913, Prob( >chi2(11)) = 5.695e-06

Variables :  Sentir_Ansioso Sentiu_deprimido
Type :       polychoric
Rho         = .68661667
S.e.        = .01444032
Goodness of fit tests:
Pearson G2 = 52.342096, Prob( >chi2(11)) = 2.364e-07
LR X2      = 49.556804, Prob( >chi2(11)) = 7.519e-07

.
```
